# Proteins associated with environmental survival of the pathogen *Neisseria meningitidis*

**DOI:** 10.1017/S0950268825100083

**Published:** 2025-06-16

**Authors:** Claire Swain, Sarah Reed, Joanna Katherine MacKichan, Thomas William Jordan

**Affiliations:** 1 Centre for Biodiscovery and School of Biological Sciences, Victoria University of Wellington, Wellington, New Zealand; 2 Institute of Environmental Science and Research (ESR), Porirua, New Zealand; 3 SCIEX, Mt Waverley, Victoria, Australia

**Keywords:** environmental survival, humidity, iTRAQ proteomics, metabolism, *Neisseria meningitidis*

## Abstract

Previously, we reported the persistence of the bacterial pathogen *Neisseria meningitidis* on fomites, indicating a potential route for environmental transmission. The current goal was to identify proteins that vary among strains of meningococci that have differing environmental survival. We carried out a proteomic analysis of two strains that differ in their potential for survival outside the host. The Group B epidemic strain NZ98/254 and Group W carriage strain H34 were cultured either at 36 °C, 5% CO_2_, and 95% relative humidity (RH) corresponding to host conditions in the nasopharynx, or at lower humidities of 22% or 30% RH at 30 °C, for which there was greater survival on fomites. For NZ98/254, the shift to lower RH and temperature was associated with increased abundance of proteins involved in metabolism, stress responses, and outer membrane components, including pili and porins. In contrast, H34 responded to lower RH by decreasing the abundance of multiple proteins, indicating that the lower viability of H34 may be linked to decreased capacity to mount core protective responses. The results provide a snapshot of bacterial proteins and metabolism that may be related to normal fitness, to the greater environmental persistence of NZ98/254 compared to H34, and potentially to differences in transmission and pathogenicity.

## Introduction

The Gram-negative bacterium *Neisseria meningitidis* is a commensal that can survive in the nasopharynx but may enter the bloodstream through invasion of the nasopharyngeal epithelia, resulting in diseases, including meningococcal meningitis and meningococcaemia [1]. It is generally considered that meningococci do not survive outside of the host; thus, the most accepted route of transmission is *via* upper respiratory tract secretions and coughing and sneezing. Our work suggests additional potential for environmental transmission from fomite surfaces [2], such as some other bacterial and viral pathogens [3, 4]. Here, we have extended our previous study of persistence of *N. meningitidis* on fomites and of differences in survival depending on isolate and environmental conditions.

Relatively little is known about how the cytoplasmic and cell wall components of bacteria contribute to persistence and transmission from surfaces. The protein-rich outer membrane of *N. meningitidis* includes porins that are involved in ion transport and differ among strains, as well as fibrous pili and transmembrane opacity proteins that are implicated in the cellular interactions and pathogenesis of the bacterium [1]. The outer and inner membranes of the cell wall sandwich a periplasmic space that includes peptidoglycan. An external polysaccharide capsule is a virulence factor that may enhance resistance to desiccation. More generally, it is known that there is substantial protein and metabolic variation among meningococcal strains, including marked differences between invasive and carriage lineages [5].

The current work is the first investigation of the molecular basis of persistence of meningococci on fomites and reports differences between two strains that differed markedly in survival. Previously, we showed that persistence was affected by temperature and humidity, including seasonal differences at ambient conditions, and was similar for invasive and carriage strains within groups, but there were marked differences in persistence between groups. Now, we have used varying culture conditions to compare the effects of humidity and temperature and analysed the protein variation using shotgun proteomics. Ideally, live bacteria retrieved from fomites would be used for this analysis, but we used iTRAQ labelling of tryptic digests of proteins from bacterial cultures to minimize safety challenges in processing proteins from multiple aliquots of the pathogen NZ98/254.

## Materials and methods

Proteins were harvested from *N. meningitidis* strains grown on Columbia Blood Agar supplemented with 5% sheep blood (Fort Richard, Auckland, New Zealand). The culture was either in an incubator at 36 °C, 5% CO_2_, 95% relative humidity (RH), or in an environmental chamber at 30 °C and 22% or 30% RH with ambient CO_2_ levels.

The epidemic strain NZ 98/254 (Type B:4:P1.7-2,4) was a patient case isolate from the New Zealand national surveillance programme. The carriage strain NZA1–478-1 (H34) (Type W:nt:P1.18-1,3) was recovered from a throat swab survey of New Zealand Army recruits. Previously reported maximum survival times on glass at 30 °C /22% RH were 196 h for NZ98/254 and 25 h for H34 [2].

Growth on Columbia Blood Agar plates was affected by the experimental conditions. Bacterial lawns formed within 18 h for NZ98/254 or H34 at 36 °C/5% CO_2_/95% RH (defined as *standard conditions* that reflect normal growth in the nasopharynx) but took 3 days for NZ98/254 at 30 °C and 22% or 30% RH, compared to 4–6 days for H34 at 30% or 22% RH. Growth of both strains was planktonic under standard conditions but was mucoid at 22% or 30% RH, possibly reflecting an increase in capsule production.

Multiplex analysis of proteins harvested from bacterial lawns was carried out using separation by strong cation exchange of tryptic digests labelled with 8-PLEX iTRAQ (isobaric tags for relative and absolute quantitation) reagents, followed by C-18 reverse-phase chromatography. Peptide spectra were collected using an AB SCIEX 5800 matrix-assisted laser desorption/ionization tandem time-of-flight mass spectrometer. The spectra were analysed using ProteinPilot v4.0.0 software (AB SCIEX) and searched against an NCBInr *Neisseria* database that contained 14 sequenced meningococcal genomes (see Supplementary Methods).

## Results

### Detected proteins

Combined analysis of 208 and 195 proteins identified in two separate technical duplicates (with 157 proteins identified in both duplicates) resulted in a total of 246 unique identifications (12% of the predicted number of meningococcal proteins). The largest number of proteins were involved in translation and ribosomal structure and function, and energy and amino acid metabolism. Eight hypothetical and four putative proteins were detected. A total of 70% of the proteins were predicted to be cytoplasmic, 6% cytoplasmic membrane, 10% outer membrane, and 5% periplasmic; others were unknown or predicted dual location (Supplementary Table S1).

### Differences between strains

Some proteins differed significantly (*p* ≤ 0.05) between the two strains grown under standard conditions at 36 °C/5% CO_2_/95% RH. Forty proteins were more abundant and 7 were less abundant in H34 compared to NZ98/254 (Supplementary Table S2). Outer membrane proteins that were more abundant in H34 included opacity protein Opa7, the surface fibrous pilus protein PilE, pilus assembly protein PilN, and twitching mobility protein PilT. Outer membrane porins PorA (GI 89276863) and PorB (GI 270728847), which have roles in ion transport and responses to osmotic stress (see below), were also more abundant in H34, likely reflecting the non-typable *porB* and P1.18-1,3 *porA* genes expressed by H34 but not by NZ98. The more abundant PorB (GI 516522) in NZ98/254 reflected the gene expressed in that strain. Several proteins associated with energy, amino acid, and lipid metabolism, or with protein folding and stress responses, were also more abundant in H34, suggesting greater metabolic responses in this strain, but there was less DNA translocase FtsK, ribonucleoside-diphosphate reductase NrdA, 50S ribosomal protein L25 (general stress protein CTC), putrescine transporters PotD-1 and PotD-3, and hypothetical protein NMB2094 (putative DNA-binding function).

### Effect of humidity and temperature on NZ98/254

Table 1 reports proteins whose abundance varied significantly between growth at 30 °C and 22% or 30% RH compared to standard conditions. Some proteins that varied between strains under standard conditions also showed altered upregulation or downregulation at altered temperature and humidity.

**Table 1.** Summary of NZ98/254 and H34 proteins that varied significantly (*p* ≤ 0.05) at 30 °C, 22% RH compared to 36 °C, 95% RH

Function	NZ98/254		H34	
	Increased at 30 °C, 22% RH	Decreased at 30 °C, 22% RH	Increased at 30 °C, 22% RH	Decreased at 30 °C, 22% RH
Cell division	FtsE^a^ ^,^^b^, MinD^a^		FtsK-2^a^ ^,^^c^	
Cell motility and secretion	PilE^a^ ^,^^b^, PilG, PilN^b^		PilE^a^ ^,^^b^	PilG^a^, PilT^a^ ^,^^b^, SecA^a^ ^,^^b^
DNA replication, recombination, and repair	RecA^a^ ^,^^b^, Ssb^a^, NMB1368			RecA^b^, Ssb
Transcription		NusA^a^, NMB0838	NusA	
Ribosomes and translation	AspS^a^ ^,^^b^, RplW, RmB^a^ ^,^^b^, TypA^a^	RpsU^a^,	AspS^b^, RplD, RplN^a^, RplP, RplR^a^, RplV, RpoC, RpsC^a^, RpsH, RpsP	AlaS^a^ ^,^^b^, RpmA^a^, RpmL^a^, RpsJ^a^, SerS^a^
Outer membrane and biogenesis cell envelope	MtrC^a^ ^,^^b^, MtrE, NlpB^a^ ^,^^b^, NlpD^a^ ^,^^b^, Omp85^a^, PorA^a^ ^,^^b^, PorB^a^ ^,^^b^ ^,^^c^, PotD–3^a^ ^,^^c^, TspA^b^, NMBNZ0533_1464^a^, NMB1468^a^ ^,^^b^, NMV1429^a^	NMBNZ0533_2030	PotD–3^c^	NlpD^b^, SiaC, TspA^b^
Energy metabolism	DipZ^a^ ^,^^b^, FixO^b^, Fpr–1^b^, PutA^b^, SucC^b^	GltA		AcnB, FixO^a^ ^,^^b^, Icd^b^, Ppa, Ppx2, PutA^a^ ^,^^b^, SdhB^a^, SucA
Amino acid metabolism and transport	GcvT^b^, IscS^a^ ^,^^b^, NMB0696	CysK^a^, GdhA^a^, GutB^a^	GdhA	DapB^a^, GcvP, GcvT^b^, GutB, IscS^a^ ^,^^b^
Carbohydrate metabolism	CbbA^a^		CbbA^a^	
Lipid metabolism	FabG2^a^ ^,^^b^			AccC^a^ ^,^^b^, FabG2^b^
Nucleotide and coenzyme metabolism	NrdA^c^, PrsA, GshA, UbiE^a^ ^,^^b^		NrdA^a^ ^,^^c^	UbiE^b^
Inorganic ion transport and metabolism				Fur^a^, NMB0378^a^, NMB0593
Signal transduction	ApaH^b^			ApaH^a^ ^,^^b^
Stress responses, chaperones, post-translational modifications	Gna1220^a^ ^,^^b^, TrxB, NMB0345^a^	GroS^a^	ClpB^b^	ClpX^b^, Gna1220^b^, GroL^a^ ^,^^b^, NMB0946^a^, NMBCU385-0690^a^
Unknown	NMBNZ0533_0109^b^			NMB1088, NMB1796, NMBNZ0533_2030

*Notes:* Proteins that differed between strains at 36 °C, 5% CO_2_, 95% RH.

a Proteins that varied similarly at 22% and 30% RH.

b More abundant in H34.

c More abundant in NZ98/254.

For NZ98/254, there were 52 proteins with significantly different abundance (43 increases and 9 decreases) at 30 °C/22% RH compared to standard conditions (Table 1 and Supplementary Table S3). Of these, 30 proteins were similarly upregulated or downregulated at 20% or 30% RH, respectively. In NZ98/254, low RH conditions resulted in an increase in proteins associated with the cell envelope and outer membrane, including membrane fusion protein MtrC, multidrug efflux pump channel protein MtrE, lipoproteins Nlp B and D, Por A and Por B, spermidine/putrescine ABC transporter PotD-3, TspA, and two hypothetical proteins. The abundance of pilus proteins, including PilE, involved in cell motility, secretion, and adhesion to host cells during invasion, also increased at 22% and 30% RH.

There were also changes in the abundance of proteins associated with stress responses, including increases in membrane protein Gna1220, thioredoxin reductase TrxB, and putative cell-binding factor NMB0345, and decreases in chaperonin GroS and cold-shock domain family protein NMB0838 (CspA). Other increases included single-stranded DNA-binding protein Ssb, recombinase RecA, and GTP-binding protein TypA, which are also associated with stress responses.

Potential metabolic responses included altered amounts of the putative amino acid transporter NMB0696, and enzymes chiefly affecting energy and amino acid metabolism, including turnover of cysteine (CysK cysteine synthase and IscS cysteine desulfurase). Single effects on CbbA (fructose-1,6-bisphosphate aldolase) and FabG2 (3-oxoacyl-[acyl-carrier-protein] reductase) indicated perturbation of carbohydrate and lipid metabolism.

In addition to the above variation at 22% RH, and at both 22% and 30% RH, 47 proteins (six increases and 41 decreases) varied only at 30% RH.

### Effect of humidity and temperature on H34

Protein changes in strain H34 were mainly decreases, with exceptions that included increased components affecting ribosomes (Table 1 and Supplementary Table S4). Major categories of protein change at 22% RH included stress responses (five decreases and one increase) and downregulation of enzymes affecting energy and amino acid metabolism. Both strains decreased the abundance of a dehydrogenase GutB at low RH, but glycine cleavage aminomethyl transferase (GcvT) and glutamate dehydrogenase (GdhA) varied differently between the strains. Decreases in proteins associated with fatty acid synthesis (acetyl-CoA carboxylase AccC and 3-oxoacyl-(acyl-carrier-protein) reductase FabG2) indicated downgraded lipid synthesis in H34, although the glycolytic enzyme fructose-bisphosphate aldolase CbbA increased at 22% and 30% RH in both strains. Changes common to both 22% RH and 30% RH were more numerous at the lower humidity. There were 18 other significant changes only at 30% RH (five increases and 13 decreases; Supplementary Table S4).

Strains 98/254 and H34, therefore, differed in their responses to altered environmental conditions. Among the few similar changes, PilE increased in both strains, although pilus proteins E, G, and N varied between strains. The putrescine/spermidine transporter Pot-D3, which has roles in cell division and growth, was upregulated at 22% RH in both strains, as were Fts family proteins that are also implicated in growth. However, there were also opposing responses; for example, DNA-processing proteins RecA and Ssb increased in NZ98/254 but decreased in H34.

## Discussion

Although environmental persistence on fomites is well established for some microbial pathogens, relatively little is known about the molecular basis of survival. One experimental approach has been to examine the effects of deletion mutants on persistence; for example, desiccation tolerance of the opportunistic pathogen *Acinetobacter baumannii* [6]. As an alternative approach, we have compared molecular variation among naturally occurring strains of *N. meningitidis.* Based on our previous results, we compared two strains that differed markedly for survival on fomites: the invasive epidemic strain NZ98/254 and a carriage strain H34, with maximum survival on glass 8 and 1 days, respectively, at 22% RH and 30 °C. Survival on fomites at 30 °C and 30% RH compared to 22% RH was approximately twofold less for NZ98/254 but did not differ between 22% and 30% RH for H34 [2]. Although these strains belonged to different capsule groups (B and W), we previously showed similar persistence of invasive and carriage strains within groups. In the current study, H34 grew more slowly in culture compared to NZ98/254. Handling of live organisms during sample preparation for proteomic analysis was minimized by using the iTRAQ method in which tryptic digestion preceded labelling and pooling of labelled samples for chromatographic separation and mass spectrometry of digested peptides.

The proteomic variation we observed is consistent with genetic and metabolic diversity between carriage and invasive strains. Schoen *et al.* [5] have suggested that environmental differences between colonization in the nasopharynx and survival in the bloodstream during invasion may be supported by metabolic specializations, including biofilm formation and nutrient limitation in the nasopharynx, and enhanced biosynthesis and preferential use of lactate as a carbon source in blood. For our analysis of differences between NZ98/254 and H34 cultured at 36 °C, 5% CO_2_, and 95% RH, 40 proteins (*p* ≤ 0.05) were more abundant in H34 (1.2- to 3.9-fold increases, including 29 proteins >1.5-fold difference) affecting mainly the outer membrane function and intermediary metabolism (Supplementary Figure S1 and Supplementary Table S2). Of the seven proteins more abundant in NZ98/254 (1.7- to 3.8-fold increases), the greatest difference was for DNA translocase FtsK, which participates in cell division. More abundant putrescine transporters PotD-1 and -3 may contribute to greater regulation of osmotic balance in NZ98/254 (see below), although the necessary functional partner PorA was more abundant in H34. All detected variation was against a background of constant levels of the other detected proteins.

### Effects of humidity and temperature

Our results provide an initial indication of proteins that are associated with survival under different environmental conditions. One qualification concerns the restricted number of proteins detected in this study (~12% of the predicted *N. meningitidis* proteome). Although both temperature and humidity were variables in our comparison, differences between 22% and 30% RH at 30 °C indicate additional effects of humidity on proteins affecting bacterial function. Others have shown differences in the formation of biofilm, auto-aggregation, and cellular adherence of *N. meningitidis* between 32 and 37 °C, accompanied by changes in the abundance of proteins from a membrane-enriched fraction of strain MC58 grown in liquid culture [7].

In the current study, survival of NZ98/254 at 22% RH was accompanied by elevation of proteins involved in cell division and DNA processing and translation, outer membrane and metabolic proteins, and stress responses (Table 1). Elevation of the cation transporter PorA is thought to facilitate entry of K+ ions to maintain osmotic homeostasis [8], accompanied by an increase of negatively charged glutamate and excretion of positively charged polyamines (by PotD-3) to maintain charge balance. In this context, an increase of PotD-3 and a decrease of GdhA, which metabolizes glutamate, suggest responses to uptake of K+ by elevated PorA in NZ98/254. Increases in Pil family proteins may be associated with enhanced twitching motility and formation of biofilms [9].

Preliminary prediction of metabolic responses suggests enhanced glycolysis (through fructose-1,6-bisphosphate aldolase CbbA), energy metabolism, and fatty acid synthesis (FabG2), but decreased flux into the citric acid cycle through citrate synthase GltA. Other proteins whose altered regulation indicated a high level of metabolic activity in NZ98/254 at 22% RH included putative amino acid transporter NMB0696, consistent with the requirement for uptake of amino acids, aminomethyltransferase GcvT, enzymes (NrdA and PrsA) required for synthesis of the precursors of nucleotide coenzymes and nucleic acids, dehydrogenase GutB, and UbiE that participates in the metabolism of quinones. Aspects of cysteine metabolism are also represented, including glutamate-cysteine ligase GshA for the synthesis of glutathione, which has multiple roles including responses to oxidative stress. Downregulation of cysteine synthase CysK accompanied by an increase in cysteine desulfurase IscS suggests depletion of cysteine that may be associated with the onset of stationary phase growth (reviewed by Schoen *et al.* [5]). Many of these proteins varied similarly at 22% and 30% RH.

In contrast, H34, which had minimal ability to survive on fomites, responded to lower humidity with decreases in protein abundance that indicate downregulation of DNA synthesis and gene expression, including ferric uptake regulation protein Fur, cell surface and outer membrane functions, and metabolic and stress responses. Citric acid cycle activity (AcnB, SdhB, and SucA) and terminal electron transport (FixO) appeared to be impaired, as was glycine (GcvP and GcvT), proline (PutA) and phosphate metabolism (Ppa, Ppx2, and NMB0378), and lipid synthesis (AccC and FabG2). We have considered that poorly surviving strains may be under metabolic stress, but that does not seem to be supported by downregulation of potential chaperonins ClpX or GroL, or of the peroxiredoxin 2 family protein/glutaredoxin NMB0946. Downregulation of sialyl transferase SiaC (polysialic acid capsule biosynthesis protein) may suggest decreased synthesis of the capsule polysaccharide. Similar to NZ98/254, the surface pilus protein PilE was upregulated at 22% and 30% RH, but unlike NZ98/54, other proteins affecting cell motility and secretion were downregulated.

### Summary and conclusions

Genetic and metabolic variation among meningococcal strains is extensive and underlies differences between carriage and invasive states. Of potential interest, therefore, may be differing responses to decreased temperature and humidity compared to differences between strains under standard conditions at 36 °C/95% RH (Table 1). Although the current comparison is limited by the restricted set of detected proteins, it appears that carriage strain H34, which has limited survival on fomites, may be upregulating proteins under standard conditions (Supplementary Table S2) that are also part of the response to environmental challenge at low RH in both H34 and NZ98/254 (see proteins with superscripts ^b^ and ^c^ in Table 1). To this extent, it appears that there may be core responses to stress, including environmental challenge, that are conceptually analogous to the proposed core metabolic differences between hyperinvasive and carriage strains [5].

One question concerns how responses in culture compare to persistence in the nasopharynx or on fomites. Our work shows similar differences between NZ98/254 and H34, including growth in culture (this study) and eightfold differences in survival on fomites [2] that are affected by environmental conditions. Both strains produced bacterial lawns within a day on Columbia Blood Agar at 36 °C, 5% CO_2_, 95% RH, but more slowly at 30 °C and decreased humidity. Slower growth was greatest for H34 at 22% RH. Metabolic responses to desiccation seem more likely on fomites, although our current results indicate metabolic, stress, and osmotic responses in NZ98/254 that are characteristic of dehydration in bacteria (reviewed by Bosch *et al.* [10]). Our results also suggest that the lower viability of H34 may be linked to a decreased capacity to mount these protective responses.

Our demonstration of strain differences and the effects of humidity and temperature on meningococcal proteins provides the basis for future study with deeper profiling of the proteome, inclusion of analysis of transcripts to explore regulatory interactions, use of biological replicates, and independent validation by measurement of individual proteins. We anticipate that these studies will be relevant to the understanding of environmental survival, transmission, and pathogenesis of meningococci and other organisms.

## Supporting information

10.1017/S0950268825100083.sm001 Swain et al. supplementary material Swain et al. supplementary material

## Data Availability

The data generated and analysed during this study are included in this published article and its Supplementary Information files.

## Abbreviations

iTRAQ: isobaric tags for relative and absolute quantitation
RH: relative humidity

## Supplementary material

The supplementary material for this article can be found at http://doi.org/10.1017/S0950268825100083.

## References

[1] Hill DJ, et al. (2010) Cellular and molecular biology of *Neisseria meningitidis* colonization and invasive disease. Clinical Science 118, 547–564. 10.1042/CS20090513. 20132098 PMC2830671

[2] Swain CL, et al. (2017) Survival of *Neisseria meningitidis* outside of the host: Environmental effects and differences among strains. Epidemiology and Infection 145, 3525–3534. 10.1017/S0950268817002473. 29103405 PMC9148751

[3] Wißmann JE, et al. (2021) Persistence of pathogens on inanimate surfaces. A narrative review. Microorganisms 9, 343. 10.3390/microorganisms9020343. 33572303 PMC7916105

[4] Katzenberger RH, Rosel A and Vonberg R-F (2021) Bacterial survival on inanimate surfaces: A field study. BMC Research Notes 14, 97. 10.1186/s13104-021-05492. 33722269 PMC7962391

[5] Schoen C, et al. (2014) Metabolism and virulence in *Neisseria meningitidis*. Frontiers in Cellular and Infection Microbiology 4, 114. 10.3389/fcimb.2014.00114. 25191646 PMC4138514

[6] Oda Y, et al. (2022) CsrA-controlled proteins reveal new dimensions of *Acinetobacter baumannii* desiccation tolerance. Journal of Bacteriology 204, e0047921. 10.1128/jb.00479-21. 35285725 PMC9017300

[7] Lappann M, et al. (2016) Impact of moderate temperature changes on Neisseria meningitidis adhesion phenotypes and proteome. Infection and Immunity 84, 3484–3495. 10.1128/IAI.00584-16. 27672084 PMC5116725

[8] Massari P, et al. (2003) The role of porins in neisserial pathogenesis and immunity. Trends in Microbiology 11, 87–93. 10.1016/s0966-842x(02)00037-9. 12598131

[9] Eriksson J, et al. (2015) Characterization of motility and piliation in pathogenic Neisseria. BMC Microbiology 15, 92. 10.1186/s12866-015-0424-6. 25925502 PMC4449605

[10] Bosch J, et al. (2021) Microbial anhydrobiosis. Environmental Microbiology 23, 6377–6390. 10.1111/1462-2920.15699. 34347349

