# Supplementary material for: Proteins associated with environmental survival of the pathogen Neisseria meningitidis
Source: Epidemiol Infect. 2025 Jun 16;153:e71. doi: 10.1017/S0950268825100083 (PMC12171898; doi:10.1017/S0950268825100083)
Supplement: Swain et al. supplementary material [file S0950268825100083sup001.docx]

Epidemiology and Infection

Proteins associated with environmental survival of the pathogen *Neisseria meningitidis*

Claire L Swain, Sarah J Reed, [Joanna K MacKichan](https://pubmed.ncbi.nlm.nih.gov/?sort=pubdate&term=Mackichan+JK&cauthor_id=29103405), T William Jordan

**Supplementary Material**

*Supplementary Figure S1*

Supplementary Figure S1. Proteins that varied in abundance between H34 and NZ98/254 under standard conditions 36°C, 5% CO_2_, 95% relative humidity. Brackets show numbers of proteins.

PilE

**More Abundant in H34**

**Gene expression (4)**

**Energy metabolism (7)**

**Other metabolism (7)**

**Cell surface/outer membrane (10)**

**Stress responses (6)**

**Other and Unknown (7)**

**More Abundant in NZ98/254 (7)**

**Includes Porins, PotD-1,-3, FtsK-2**

**Less Abundant in H34**

*Supplementary Tables*

Supplementary Table S1. Identified proteins and analysis of variation between conditions. The Table summarizes combined results from the two technical replicates, with statistical analysis of protein differences at 30^o^C and 22% or 30% RH, compared to strain NZ98/254 at standard conditions at 36 ^o^C and 95% RH.

Columns P-AC are Log_2_ ratios. Cells highlighted in yellow indicate greater than 1.8-fold increase in abundance. Blue cells indicate abundance less than 0.2-fold. Green cells indicate a significance of greater than 95%. Lilac cells indicate a significance between 90% and 94.99% confidence. Empty ratio cells indicate that insufficient quantitation data was obtained for that protein. For multiplex labelling design see Supplementary Methods.

The Tabs contain the underlying detail of individual results for the two experiments, with protein ratios based on comparison with either NZ98/254 (NZ98i) or H34 (H34i).

Supplementary Table S2. Proteins that varied between H34 and NZ98/254 at 36 ^o^C and 95% RH. NZ98i are values for NZ98/254. Combined results from two experiments.

Supplementary Table S3. NZ98/254 proteins that varied at 30 ^o^C and 22% or 30% RH compared to 36 ^o^C and 95% RH (NZ98i). Combined results from two experiments.

Supplementary Table S4. H34 proteins that varied at 30 ^o^C and 22% or 30% RH compared to H34 at 36 ^o^C and 95% RH (H34i). Combined results from two experiments.

Supplementary Tables S1-S4 Footnotes

1 = Gene name equivalent to serogroup B Neisseria meningitidis (NMB) as per BLAST search; 2 = The equivalent NMB protein name, which is MC58 unless otherwise stated and is the same protein name as original unless otherwise stated. The shared measure of identity of the equivalent NMB protein with the non-meningococcal protein is given in the ‘Gene Name NMB’ column using superscript letters.

COG function codes: Information, storage and processing – J = Translation, ribosomal structure and biogenesis, K = Transcription, L = DNA replication, recombination and repair; Cellular processes – D = Cell division and chromosome partitioning, M = Cell envelope biogenesis, outer membrane, N = Cell motility and secretion, O = Posttranslational modification, protein turnover, chaperones, P = Inorganic ion transport and metabolism, T = signal transduction mechanisms; Metabolism – C = Energy production and conversion, E = Amino acid transport and metabolism, F = Nucleotide transport and metabolism, G = Carbohydrate transport and metabolism, H = Coenzyme transport and metabolism, I = Lipid metabolism; Q = Secondary metabolites biosynthesis, transport, and catabolism; Poorly characterised proteins – R = General function prediction only, S = Function Unknown, No Related COG = Protein does not match to any COG.

Subcellular location codes: C = cytoplasm, CM = Cytoplasmic membrane, P = Periplasm, OM = Outer membrane, E = Extracellular, UM = Unknown due to multiple possible locations, U = Unknown

*Supplementary Methods*

Culture

Proteins were harvested from *N. meningitidis* strains grown on Columbia Blood Agar supplemented with 5% Sheep Blood (Fort Richard, Auckland, New Zealand) either in an incubator at 36°C, 5% CO_2_, 95% relative humidity (RH) or in environmental chamber at 30°C and 22% or 30% RH (Swain *et al.* 2017). All live meningococcal work was undertaken in the Invasive Pathogens and Meningococcal Vaccine Antibody Testing laboratories, ESR, Kenepuru, Wellington.

Proteomic strategy

Quantitative analysis of proteins was carried out using separation by strong cation-exchange (SCX) of tryptic digests labelled with iTRAQ regents followed by C-18 reverse phase liquid chromatography and MALDI-TOF/TOF mass spectrometry (LC-MS/MS).

All results are derived from single cultures for each of the strains and environmental conditions. The protocol required reduction, alkylation, tryptic digestion and labelling of each individual sample followed by pooling and SCX and LC-MS/MS of the pooled samples. Each culture was analysed in two separate experiments (Experiments 1 and 2) and in each experiment the pooled samples was analysed twice using the offline chromatography – mass spectrometry protocol.

Tryptic digestion of proteins and labelling of the peptides

Bacteria harvested in 1 mL phosphate buffered saline pH 7.4 (PBS) were washed three times with PBS with centrifugation for 3 min at 13,000 rpm. Washed pellets were re-suspended in NuPAGE LDS buffer (Thermo Fisher Scientific, Waltham, MA) with 20 mM dithiothreitol and protease inhibitor cocktail (Roche 046931590001, Sigma Aldrich, St. Louis, MO), then heat-killed for 1 h at 56°C. Samples containing 400 µg protein were then precipitated using a Calbiochem ProteoExtract Protein Precipitation Kit (Merck, KgaA, Darmstadt, Germany) and resuspended in 50 µl Dissolution Buffer (iTRAQ Reagent Multiplex Buffer Kit, AB SCIEX #4390812, Framingham, MA). Aliquots containing 100 µg protein were reduced with dithiothreitol, alkylated with iodoacetamide, and digested with trypsin at a protein: trypsin ratio 50:1 for 16 h at 37 °C. Samples were then reacted with reagents from the 8plex amine modifying iTRAQ kit so that individual samples were labelled for 2 h at room temperature with either 113, 114, 115, 116, 117, 118, 119 or 121 isobaric mass tags.

## iTRAQ Labelling Design

iTRAQ analysis of differences in protein amount was carried out on NZ98/254 and H34 meningococcal strains grown either in an incubator at 36°C, 5% CO_2_, 95% RH, or in an environmental chamber at 30°C and 22% or 30% RH. The three conditions for each strain, plus two pooled controls, were assigned to one of the eight iTRAQ labels available for the experiment. Pooled controls consisting of equal measures of the six samples were used as internal replicates to identify the level of intra-sample variation. A comparison of the protein expression level differences between the two controls using a Volcano plot was used to determine the appropriate P-value threshold required to reduce the number of differential expression false positives.

The iTRAQ labelling design was:

|  | **iTRAQ Labels** | | | | | | | |
| --- | --- | --- | --- | --- | --- | --- | --- | --- |
|  | **113** | **114** | **115** | **116** | **117** | **118** | **119** | **121** |
| **Sample** | NZ98/254 Standard conditions* | H34 22% RH | H34 30% RH | NZ98/254 22% RH | H34 Standard conditions* | Pooled control | Pooled control | NZ98/254 30% RH |

*Standard conditions. Incubator 36°C, 5% CO_2_, 95% RH. All other cultures were at 30°C and 22% or 30% RH.

Separation and mass spectrometry of labelled peptides

The iTRAQ-labelled tryptic digests were pooled to provide composite samples, containing aliquots of each of the two strains and three culture conditions, for chromatographic fractionation by SCX on an AB SCIEX iCAT column, (#4326695) using elution at 50, 100, 150, 200, and 350 mM KCL. Peptides from the five SCX fractions were further separated using a Tempo LC MALDI spotting system (AB SCIEX) equipped with a 150 mm x 100 µm Monolithic Chromolith® CapRod® Reverse Phase C-18 endcapped column (Merck Millipore, Billerica, MA) using a 100 min gradient from 2-80% acetonitrile in 0.1% trifluoroacetic acid. Eluted peptides mixed 1:1 with a saturated CHCA (alpha-cyano-4-hydroxycinnamic acid) solution in 50% acetonitrile - 0.1% trifluoroacetic acid were spotted in triplicate directly onto Opti-TOF LC/MALDI 123 mm x 81 mm stainless steel plates (AB SCIEX).

Identification of proteins

The peptide spectra were collected using an AB SCIEX 5800 MALDI TOF/TOF mass spectrometer as previously described (Dunne *et al*. 2015). The TOF/TOF files were analysed using ProteinPilot v4.0.0 software (AB SCIEX) (Seymour and Hunter, 2018) and searched against the NCBInr *Neisseria* database that contained 14 sequenced meningococcal genomes (*Neisseria* 249626 sequences, *N. meningitidis* 39,508 sequences, 9 November 2011). Each sample was analysed by LC-MS/MS twice and the resulting spectra were combined using the Paragon method in Protein Pilot.

Search parameters were iTRAQ-8plex, cysteine carbamidomethylation as a fixed modification, methionine oxidation as a variable modification, maximum one missed trypsin cleavage, maximum 50 ppm and 0.05 Da mass tolerances for MS and MS/MS spectra respectively. Proteins were accepted as detected at confidence threshold ≥ 99% (Unused ProtScore ≥ 2.0) and false-discovery rate ≤1%. Proteins matching serogroup B *N. meningitidis* strains (preferably MC58 or NZ05/33, otherwise H44/76) were substituted into the results tables even if the best match was to another strain. BLASTp searches were carried out for proteins that did not have meningococcal equivalent to obtain the MC58 or other equivalent. Identified proteins were classified into functional groups using COGnitor (www.ncbi.nlm.nih.gov/COG) based on Clusters of Orthologous Groups (COGs). PSORTb v 3.0 (www.psort.org/psortb/) was used to predict subcellular locations of proteins.

The results of two separate analyses (Experiments 1 and 2) were combined (total 403 protein entries) and refined using Batch Entrez (<https://www.ncbi.nlm.nih.gov/sites/batchentrez>) to produce a composite protein list. Removal of 76 duplicates based on Gi number resulted in a list of 325 “unique identifications” that included some further duplicates based on inspection of protein names. Manual removal of the name duplicates resulted in a final list of 246 proteins.

Quantitative analysis

Quantitative analysis of variation was carried out using proprietary software in ProteinPilot that computes p-values based on average ratios from the iTRAQ mass signals calculated for each protein at each of the experimental conditions. Cut-offs were calculated using iTRAQ technical replicates with 45 or 90 µg protein (n=4 each) from NZ98/254 grown at 36°C, 5% CO_2_, 95% RH. Amounts of individual proteins were significantly different (p ≤0.05) for variation ≥ 80% (≤ 0.2 or ≥ 1.8-fold). Comparisons of differences in abundance of individual proteins between samples are reported as log_2_ ratios.

References

Dunne JC, Lamb DS, Delahunt B, Murray J, Bethwaite P, Ferguson P, Nacey JN, Sondhauss S, Jordan TW. Proteins from formalin-fixed paraffin-embedded prostate cancer sections that predict the risk of metastatic disease. Clin Proteomics. 2015 Sep 16;12(1):24. doi: 10.1186/s12014-015-9096-3. eCollection 2015

Seymour SL, Hunter CL. ProteinPilot^TM^ Software overview. (<Https://sciex.com/tech-notes/life-science-research/proteomics/proteinpilot--software-overview>) Accessed 21 March 2025.

Swain CL, Martin DR, Sim D, Jordan TW, Mackichan JK. Survival of *Neisseria meningitidis* outside of the host: environmental effects and differences among strains. Epidemiol Infect. 2017 Feb;135(2):315-20. doi: 10.1017/S0950268806006789
